# The interplay between local biodiversity floral odours and reproductive success varies across different population sizes of *Cypripedium calceolus*

**DOI:** 10.1098/rsos.250207

**Published:** 2025-08-20

**Authors:** Melissa Haouzi, Olivia Rusconi, Rahel Boss, Christophe J. Praz, Gregory Röder, Théo Steiner, Sergio Rasmann

**Affiliations:** ^1^ Institute of Biology, University of Neuchâtel, Neuchâtel, Switzerland

**Keywords:** plant conservation biology, deceptive pollination, Red List species, solitary bees

## Abstract

To attract pollinators, individuals of the threatened and food-deceptive orchid *Cypripedium calceolus* (L.) employ visual and olfactory signals, notably volatile organic compounds (VOCs). However, habitat fragmentation has disrupted its population into patches of still relatively large sizes, but also small sizes. We hypothesized that small orchid populations inhabit low-biodiversity areas with fewer pollinators, potentially leading to extinction. To investigate whether local biodiversity and olfactory signals vary with population size, we analysed site-specific vegetation characteristics, insect and flower visitor diversity, plant growth traits and floral VOC profiles in small and large *C. calceolus* populations. Our results revealed that smaller populations occupy habitats with lower biodiversity, consist of smaller plants with fewer flowers and exhibit reduced flowering success compared to larger populations. VOC profiles also varied between population sizes. However, fruit production and reproductive success did not differ, indicating that these chemical and ecological differences do not necessarily affect reproductive output. These results highlight that population size is linked to variation in plant traits and floral scent but does not directly predict reproductive success under current conditions.

## 1. Background

The fate of flowering plants and their pollinators is intrinsically coupled [1,2]. As most flowering plants depend on pollinators to maintain their reproductive fitness [3,4], biotic or abiotic factors that affect one are likely to affect the other [2,5]. In this regard, the unprecedented loss of biodiversity that’s occurring worldwide [6] is not only causing losses of plants and insects at the individual level but also causing changes in the processes underlying the interactions between community interactions and ecosystem functioning [7–11]. Although biodiversity loss is expected to have widespread repercussions across all plant species [12], its impacts are likely to be especially severe for rare and threatened species. These species often inhabit fragmented habitats that are largely isolated from extensive pollinator networks [13,14]. Nevertheless, there is a paucity of studies investigating how rare plants sustain their fitness despite the presence of small and fluctuating pollinator communities [4,15].

To attract pollinators to the reproductive organs, flowering plants have developed a wide range of functional traits that include visual and olfactory signals [16,17]. For instance, plants produce floral volatile organic compounds (VOCs) [15] that can attract pollinators or act as repellents when florivores are present [18–20]. More than 1700 individual floral VOCs have been found in the plant kingdom, and countless combinations of them are emitted by flowers [21]. Such odour blends have been shown to vary across and within species [22–25], a variability most likely to be driven by both genetic and environmental variation [26]. Additionally, floral scents can vary at different levels, whether in terms of overall composition, relative quantities or absolute quantities [27,28]. However, while the role played by floral scents in the attraction of pollinators is becoming increasingly recognized, their role in reproductive fitness for rare endangered plant species remains largely unknown [15,29].

A species that has been shown to also use VOCs to attract pollinators is the rare and emblematic orchid *Cypripedium calceolus* L. or lady’s slipper (Orchidaceae) [30]. While the species is distributed from the United Kingdom to the Pacific Ocean [30], the number and size of its populations have drastically declined over the last decades, with the species now being listed in the International Union for the Conservation of Nature (IUCN) Red List as threatened or critically endangered in multiple countries [31–33]. In addition to overharvesting and habitat destruction, *C. calceolus* disappearance is linked to its biology and ecological interactions [34]. *C. calceolus* plants are characterized by slow development time and long life expectancy, potentially living more than 30 years. The first green leaves only appear between one and four years after germination, while flowering occurs 6–16 years after germination, making reintroduction attempts arduous and mostly unsuccessful so far [35]. *C. calceolus* can reproduce vegetatively or sexually. Vegetative reproduction occurs through the production of tufts of clones from rhizome ramifications. Sexual reproduction occurs via insect pollination, with insects, mostly solitary bees and flies, being lured to enter the *C. calceolus* labellum by both odour and visual signals [36–38]. Indeed, *C. calceolus* orchids use a food-deceptive pollination system, in which flowers advertise, but do not provide rewards (e.g. nectar) [39]. Once inside the labellum, the rewardless pollinators attempt to escape the floral trap via the posterior exit, where pollinia are deposited onto their bodies [38]. Such a mode of reproduction might not directly facilitate the spread of this species, as it often involves intricate interactions between orchids and their pollinators, including mimicry, chemical deception and specific pollinator behaviours that vary across species and environments.

Accordingly, non-rewarding flowers must adopt various strategies to attract pollinators and optimize their reproductive success in response to environmental conditions [40,41]. For instance, an adaptive process has been demonstrated in the *Disa ferruginea* orchid, where variations in flower colour between different localities align with local pollinator preferences, suggesting the presence of ‘floral Batesian mimicry’ [42]. Similarly, a pollinator-rich environment can significantly benefit deceptive species, such as the *Anacamptis morio* orchid, by promoting pollinator recruitment [43]. This phenomenon, known as the ‘magnet species effects’, enables rewarding species to boost the pollination success of neighbouring non-rewarding plants [44]. Therefore, the surrounding environment plays a crucial role in shaping the attractiveness of floral signals to ensure successful pollination. The erosion of biodiversity, which can impact the environment quality and the size of populations, can therefore affect the various strategies that plants develop in order to ensure their reproductive fitness.

Within this context, it can be hypothesized that plants from populations of varying sizes are embedded into distinct local environmental conditions, which also probably differ in terms of vegetation composition and entomofaunal diversity. In turn, such abiotic and biotic differences should affect plant traits-pollinator interaction, and ultimately, reproductive success [45]. To explore these complex connections between population size, local biodiversity and plant functional traits, we studied multiple *C. calceolus* individuals originating from large and small populations in Switzerland. We specifically asked the following questions. (i) Are large populations of *C. calceolus* found in more biodiverse sites, substantiating a potential link between rare species decline and biodiversity loss? (ii) Does pollinator abundance favour higher reproductive success? (iii) Do individual orchids from populations of different sizes exhibit chemical differences, influencing chemical communication? (iv) Do *C. calceolus* mimic floral VOCS of the most abundant co-flowering species to optimize their reproductive success? Notably, we observed that *Hieracium murorum* aggr. is a predominant nectar source for solitary bees in forested ecosystems where *C. calceolus* is also found [46], and these bees are also the main pollinators of *C. calceolus* [23,24,38]. Given the similarity in flower colour and shared pollinator species, *H. murorum* serves as an ideal model to test for floral Batesian mimicry.

## 2. Methods

### 2.1. Site selection and vegetation monitoring

**Figure 1.**
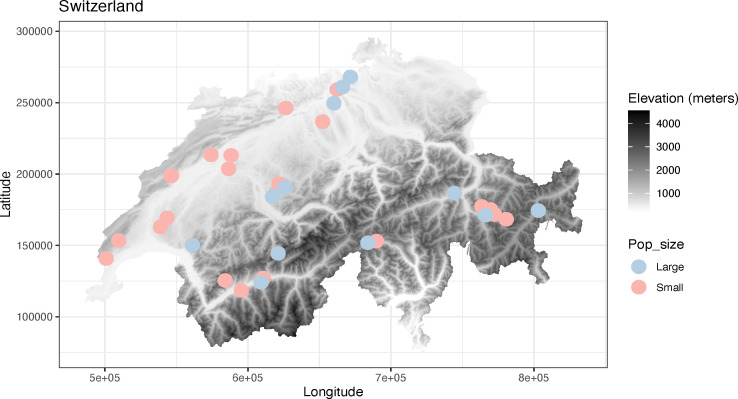
Experimental design of the study. Elevation map of Switzerland with the 34 sampled populations of *Cypripedium calceolus*. Large populations are represented by a blue dot, while small populations are represented by a red dot.

We randomly selected 34 *C*. *calceolus* populations throughout Switzerland based on known occurrences, provided by the national data and information centre on Swiss flora (https://www.infoflora.ch/en/) (figure 1). Additional selection criteria included site accessibility, possibility of obtaining visitation permits, and population size. We specifically categorized populations as being small (i.e. less than 20 inventoried individuals per population) or large (i.e. more than 100 individuals per population). We considered each stem or group of stems (patch) separated by a minimum of 70 cm from each other as an individual plant [30]. Therefore, we selected 22 small (*n* < 20) and 12 large (*n* > 100) populations across Switzerland (electronic supplementary material, table S1, figure 1). Each population was visited between 2017 and 2021, once during the peak flowering period, between April and June, and once after fruit (i.e. seed pods) production, between July and August. At each site, we performed a vegetation survey on a 10 × 10 m^2^ plot placed in the centre of the *C. calceolus* population to characterize local plant diversity. Vegetation surveys were done in the most homogenous vegetation type following the Braun–Blanquet method [47]. Based on the vegetation community structure (characteristic and most abundant species), we assigned an alliance name to each vegetation type based on phytosociological nomenclature according to Delarze *et al*. [48] (see electronic supplementary material, table S1).

### 2.2. Insect and pollinator survey

*Local insect biodiversity survey*. To obtain a snapshot of the flying insects’ diversity during flowering, we installed four yellow pan traps filled with water and a drop of soap around *C. calceolus* individuals chosen randomly during their blooming period (electronic supplementary material, figure S1) [49]. The traps were separated from each other by a minimum of 2 m. Insect sampling was performed on sunny days, and for each population, plates were installed at 9 a.m. and removed at 4 p.m. Trapped insects were washed from the soapy solution and stored in 70% EtOH. Potential pollinators present in each population, specifically bees and syrphid flies found in the traps [24,38,50], were identified to the species level and their sex was determined. All other insects were identified to the morphospecies level [51,52].

*Flower-visiting insect survey*. To determine the insects that visit the labellum of *C. calceolus* during the blooming period, we performed an experiment as described by Case & Bradford [53] on 10 (or the maximum number present if less than 10) individuals per population. We used the natural trapping system of *C. calceolus* and blocked its exit with a yellow ribbon. By doing so, we were able to catch the insect trapped in the flower by taking off the ribbon, positioning a net around the flower, and waiting until the insect emerged from the labellum into the net. We attributed each insect to one of the two following categories: (i) without pollinia, or (ii) with pollinia. All captured bees and syrphid flies were identified to the species level and their sex was determined. The other insects were again identified to morphospecies.

### 2.3. Plant traits

For all sites, on each individual of *C. calceolus* (*n* = 2–10 depending on population size), we measured functional traits with established relationships to fitness and reproduction [45,54]. We randomly chose patch-forming individuals within a 100 m^2^ area, whereby selected individuals were separated by a minimum of 2 m. We measured: (i) clonal patch area, measured as an ellipse circumscribing the individual patch (cm^2^); (ii) number of flowers per patch (measured during the first visit); (iii) flowering success for each individual, estimated as the total number of flowers divided by the total number of stems; (iv) number of fruits (pods) per individual (during the second visit); and (v) reproductive success for each individual, estimated as the total number of fruits per individual divided by the number of flowers per individual in each patch.

### 2.4. Collection of flower VOCs

Flower VOCs were collected following the protocol of Braunschmid *et al*. [23], and applying a dynamic headspace collection and analysis method as described in Dötterl *et al*. [27], with some modifications (electronic supplementary material, figure S2). Specifically, we enclosed individual flowers in a nalophan oven bag (Tangan no. 34, 35 × 40 cm, Migros-Genossenschafts-Bund, Switzerland) fixed to a bamboo stick and closed the bag with a cord around the stalk. After 15 min, we punched a hole in the plastic bag and fixed a plastic pipe (Tecnotubi Picena Srl, 4 mm inner diameter, 40 cm long) connected to an adsorbent glass tube filled with Tenax-TA (GERSTEL GmbH & Co., Buchem B.V., The Netherlands), itself plugged using a Teflon glass connector. Because VOCs are extremely volatile, thermal desorption was chosen for its low detection limit. The headspace around the flower was then passed through the Tenax tube for 15 min (200 ml min^−1^) with an AirChek 52 Sample Pump (SKC Company, Chino Hills, CA, USA). In each population, we also included a ‘blank’ by collecting air contained inside an empty nalophan oven bag fixed to a bamboo stick. Tenax tubes were transported to the laboratory at 4°C and kept at −80°C until analysis. To identify if *C. calceolus* mimic odour from surrounding co-flowering vegetation, we additionally sampled VOCs from *H. murorum* aggr., when available. As shown in electronic supplementary material, table S2, we found several co-flowering species across the field campaign. From these, we selected *H. murorum* because it was the most common co-flowering species that produces yellow-coloured flowers across all sites during the flowering of *C. calceolus* and that can also be visited by short-tongued bees, which are known to be the primary and most effective pollinators of C. calceolus due to their size and foraging behaviour.

### 2.5. Analysis of flower VOCs

Chemical samples were analysed using an automatic thermal desorption system coupled to a Gas Chromatograph and a Mass Spectrometer (GC-MS) (GC: Agilent 7890A, MS: Agilent 5975C, Agilent, Santa Clara, CA, USA). An internal standard (1 μl of naphthalene in dichloromethane; 20 ng µl^−1^) was added to each sample immediately prior to analysis. Tenax tubes were desorbed for 3 min (initial temperature: 50°C, hold time 0.25 min, ramp 60 °C min^−1^ until 250°C), then cryofocused in the Cooled Injection System (CIS) at −80°C (Programmable Temperature Vaporizing solvent vent mode, 250°C, 14 psi pressure, 50 ml min^−1^ purge and vent flows, helium as carrier gas). After desorption, the temperature in the CIS was rapidly increased to 260°C (rate 12 °C s^−1^) and compounds were transferred into a capillary column (HP−5MS, 5% phenyl methyl silox, 30 m long, 250 μm diameter, 0.25 μm film thickness) for separation, using helium as the carrier gas (constant flow mode at 0.9 ml min^−1^). The GC program started at a temperature of 50°C for 30 s, increased 5 °C min^−1^ to 250°C, stabilized at 250°C for 1 min and had a post-run at 260°C for 1 min. The transfer line temperature to the mass spectrometer was set at 280°C, before reaching a mass detector (Electron Impact mode, 70 eV) set to scan masses ranging from m/z values of 33–350.

The entire VOCs matrix was aligned based on retention time and ion spectra using MZmine v2.53 software [55]. Identification of individual compounds was done thanks to their mass spectra and fragmentation analyses comparisons with pure standards and with published spectra in the NIST05 database. However, two sesquiterpenes remained non-annotated (table 1). Relative quantities without the internal standard and absolute quantities as a function of the internal standard (naphthalene) were calculated. For statistical analyses, because samples were collected across three seasons, and across a wide variety of habitats, we chose the conservative approach of only retaining those VOCs that were present in at least 3 individuals across all years (table 1).

**Table 1.** Volatiles organic compounds of *Cypripedium calceolus*. Proportions (means ± s.e.) in small and large populations for each chemical compound analysed in this study.

name of compound	small populations	large populations
octanal	0.194 ± 0.057	0.201 ± 0.088
(Z)-hexenol-acetate	0.749 ± 0.263	1.902 ± 0.592
hexyl acetate	3.784 ± 0.561	4.124 ± 0.711
limonene	0.563 ± 0.229	0.962 ± 0.559
octanol	0.993 ± 0.212	0.292 ± 0.091
linalool	14.016 ± 1.019	18.078 ± 2.289
nonanal	4.431 ± 0.735	5.602 ± 1.473
2-phenylethanol	1.064 ± 0.216	1.296 ± 0.466
4-oxoisophorone	2.091 ± 0.488	2.815 ± 1.341
benzyl acetate	2.981 ± 1.322	5.558 ± 2.340
decanal	1.162 ± 0.268	1.847 ± 0.672
octyl acetate	54.472 ± 2.281	44.717 ± 4.049
2-phenethyl-acetate	2.959 ± 0.677	2.818 ± 1.036
(Z)−7-hexadecenal	0.059 ± 0.020	0.099 ± 0.048
tetradecene	0.080 ± 0.024	0.343 ± 0.115
decyl acetate	5.879 ± 0.713	3.461 ± 0.606
alpha-farnesene	0.144 ± 0.038	0.227 ± 0.095
U-sesquiterpene1	0.056 ± 0.029	0.339 ± 0.242
pentanoic acid	0.608 ± 0.174	1.929 ± 0.622
U-sesquiterpene2	0.055 ± 0.021	0.259 ± 0.116
lauryl acrylate	3.662 ± 1.187	3.130 ± 1.582

### 2.6. Data analysis

All statistical analyses were performed with R software (v4.3.3) [56].

*Diversity of plants and insects*. For both vegetation and insect datasets, we calculated three indices of community diversity: (i) species richness; (ii) Shannon diversity index; and (iii) total abundance of individuals. The Shannon diversity index was calculated using the *diversity* function from the *vegan* package (v2.6-4) [57]. Only insects identified as potential pollinators were included in these analyses, specifically Hymenoptera and Diptera. Next, we assessed the effect of population size (two levels, i.e. small and large) on each index, individually, using general linear models (GLM), with population size as the explanatory variable and quasi-Poisson distributions. ANOVAs based on these models were performed using the *car* package (v3.1−2) [58]. Additionally, to compare the proportion of males and females trapped in the labellum, binomial tests were conducted for Hymenopterans and Dipterans insects.

*Plant traits*. We evaluated the effect of population size (two levels, i.e. small and large) on patch size, number of flowers, flowering success, number of fruits and reproductive success using ANOVAs with chi-square tests in GLM frameworks. In each model, quasi-Poisson distributions were used except for the reproductive success, for which the quasi-binomial distribution was used. Since multiple individuals were sampled per site, site was included as a fixed-effect blocking factor in all analyses.

*Flower VOCs*. First, we assessed effects of *C. calceolus* population size (two levels, i.e. small or large) and site on the entire volatile matrix with a permutational analysis of variance (PERMANOVA, *n* = 999 permutations) with the *adonis* function from the *vegan* package (v2.6−4) [57]. The Bray–Curtis distance metric was applied to calculate the dissimilarities in the relative amounts of each compound per flower. Chemical composition dissimilarities between samples were illustrated with a two-dimensional principal coordinate analysis (PCoA) performed using the *ape* package (v5.6-2) [59]. Then, to identify which chemical compounds contribute most to the observed variance between small and large populations, a similarity percentages (SIMPER) test was performed. The mean proportions of each compound between small and large population were represented with a mirror barplot.

Given that we did not observe any difference between small and large populations in terms of reproductive success, but that we did observe differences in chemical compositions (see §3 below), we further assessed the relative importance of chemical compounds for reproductive success without taking population size into account. For this, we used random forest, a machine learning algorithm that can determine the importance of different features in a dataset using Gini importance, using the *rfPermute* function from the *rfPermute* package (v.2.5.2) [60].

Finally, we compared the VOC profiles emitted by *H. murorum* to those emitted by all *C. calceolus* flowers sampled using a PCoA based on Bray–Curtis distances, as described above. The effect of flower species on the total chemical compositions was tested with a PERMANOVA test, as previously described.

## 3. Results

### 3.1. Diversity of plants and insects

**Figure 2.**
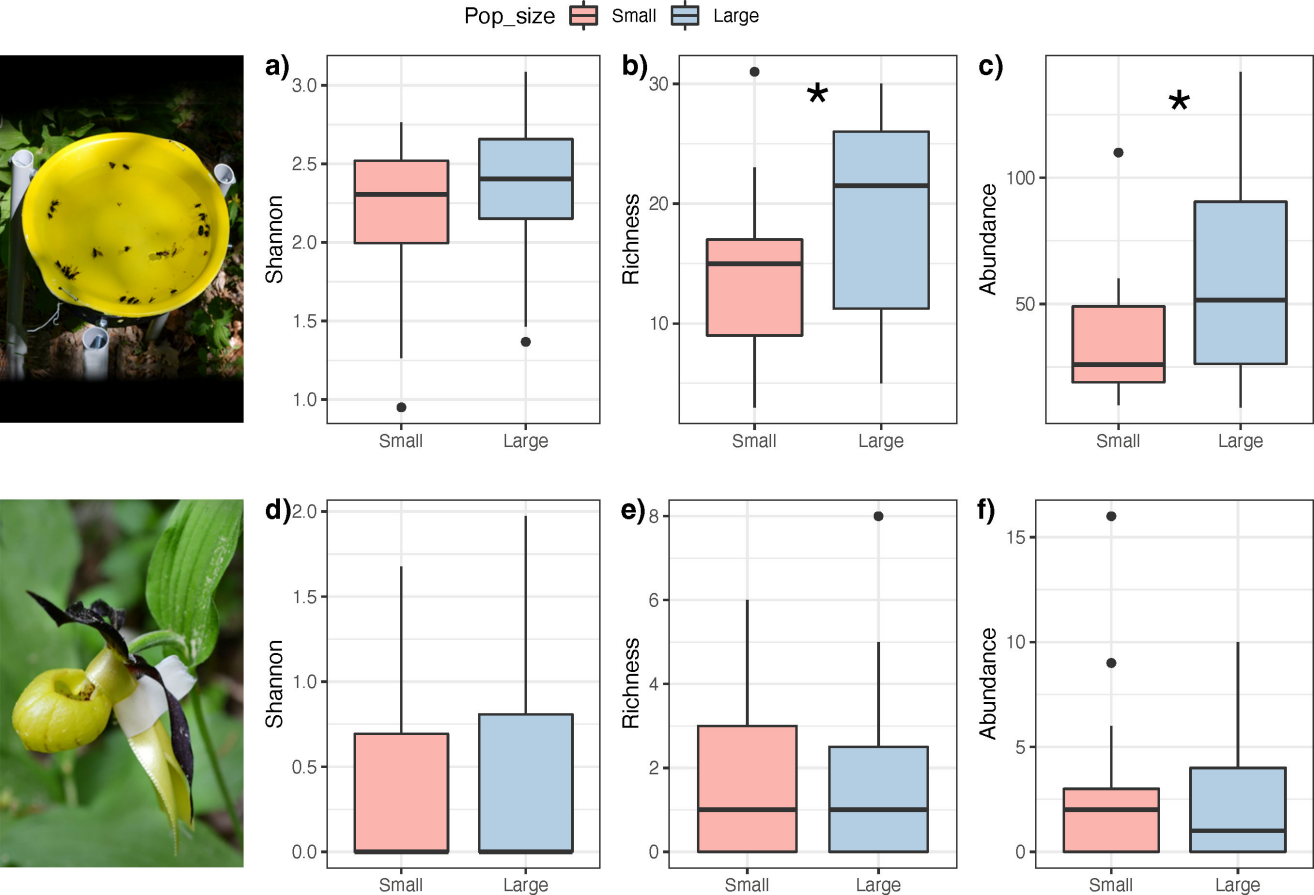
Insect biodiversity survey. Boxplots of the different biodiversity indices for (a–c) insects collected using pan traps and (d–e) insects trapped in *Cypripedium calceolus* labellum in small (<20 individuals, red boxplots) and large (>100 individuals, blue boxplots) populations. (a,d) Shannon diversity, (b,e) species richness and (c,f) total abundance. Boxplots illustrate the median (middle bar) and the range between 1st and 3rd quartiles with the whiskers extending to the upper and lower inner values. Asterisks indicate significant differences across population sizes (*p* < 0.05).

We found that *C. calceolus* populations were associated with 8 different vegetation alliances and a species richness ranging from 16 to 60 species per site (electronic supplementary material, table S1). Small and large populations of *C. calceolus* were found in distinct plant communities that differ in biodiversity. Large populations were surrounded by plant communities with a Shannon diversity index that is 11.30% higher than those surrounding small populations (electronic supplementary material, figure S3a; chi-squared = 4.692, *p* = 0.030). We found no effect of population size on either plant species richness (electronic supplementary material, figure S3b; chi-squared = 1.487, *p* = 0.223) or total vegetation abundance (electronic supplementary material, figure S3c; chi-squared = 2.315, *p* = 0.128). We also found that large and small *C. calceolus* populations are associated with insect communities that differ in biodiversity. Specifically, while we found weak effects on Shannon diversity (figure 2a; Shannon index: chi-squared = 3.407, *p* = 0.065), we found that large populations are surrounded by insect communities that are in average 74.33% more abundant (figure 2c; chi-squared = 4.614, *p* = 0.031), and that are 56.23% more species-rich (figure 2b; chi-squared = 8.055, *p* = 0.004) than small populations.

On the other hand, we found no differences in the Shannon diversity (figure 2d; chi-squared = 0.034, *p* = 0.854), species richness (figure 2e; chi-squared = 0.111, *p* = 0.738), or total abundance (figure 2f; chi-squared = 0.068, *p* = 0.795) for the insects that were observed being trapped in the labellum across small and large populations. That said, throughout the field campaign, we observed a total of 27 different species of Hymenoptera and Diptera in *C. calceolus* labella (table 2). Overall, we found many more females than males visiting the labellum without carrying pollinia (electronic supplementary material, figures S4; 6 females/6 dipteran (*p* = 0.015), and 54 females/62 hymenopteran (*p* < 0.001)). Thirteen species (represented by 25 individuals) had pollinia on their thorax when they exited *C. calceolus* labellum and were considered as pollinators (table 2). Among them, we found six Hymenopteran and one Dipteran species, which, to our knowledge, represent novel pollinator records for *C. calceolus*: *Andrena fulvata*, *Andrena strohmella*,* Lasioglossum lativentre*,* Lasioglossum nitidulum*,* Lasioglossum pygmaeum*,* Monostegia abdominalis* and *Melanostoma scalare*.

**Table 2.** Insect pollinators of *Cypripedium calceolus*. The table describes all the insect species found in the labellum of 34 Switzerland populations of *C. calceolus*. It indicates the number of observations per species (*n*), the number of those observations in which the insect carried pollinia on their torso, whether this was a first record of such an event (in bold and with superscript **^N^**) and which order they belong to (Hymenoptera or Diptera).

species	*n*	pollinia	insect order
*Andrena bicolor*	3	1	Hymenoptera
***Andrena bucephala* ^N^**	1	0	Hymenoptera
***Andrena fulvata* ^N^**	4	2	Hymenoptera
*Andrena haemorrhoa*	2	1	Hymenoptera
*Andrena nigroaenea*	1	1	Hymenoptera
*Andrena rogenhoferi*	4	0	Hymenoptera
*Andrena scotica*	6	0	Hymenoptera
***Andrena strohmella* ^N^**	1	1	Hymenoptera
***Cheilosia flavipes* ^N^**	1	0	Diptera
***Cheilosia proxima* ^N^**	1	0	Diptera
***Cheilosia rhynchops* ^N^**	1	0	Diptera
***Dasysyrphus lenensis* ^N^**	2	0	Diptera
*Halictus tumulorum*	2	1	Hymenoptera
*Lasioglossum albipes*	7	0	Hymenoptera
***Lasioglossum bluethgeni* ^N^**	4	0	Hymenoptera
*Lasioglossum calceatum*	6	5	Hymenoptera
***Lasioglossum fulvata* ^N^**	1	0	Hymenoptera
***Lasioglossum lativentre* ^N^**	1	1	Hymenoptera
***Lasioglossum minutulum* ^N^**	1	0	Hymenoptera
*Lasioglossum morio*	1	1	Hymenoptera
***Lasioglossum nitidulum* ^N^**	15	6	Hymenoptera
***Lasioglossum parvulum* ^N^**	1	0	Hymenoptera
***Lasioglossum pygmaeum* ^N^**	2	1	Hymenoptera
*Melanostoma mellinum*	2	0	Hymenoptera
***Melanostoma scalare* ^N^**	3	3	Diptera
***Platycheirus parmatus* ^N^**	1	0	Diptera
***Monostegia abdominalis* ^N^**	1	1	Hymenoptera

### 3.2. Plant traits

**Figure 3.**
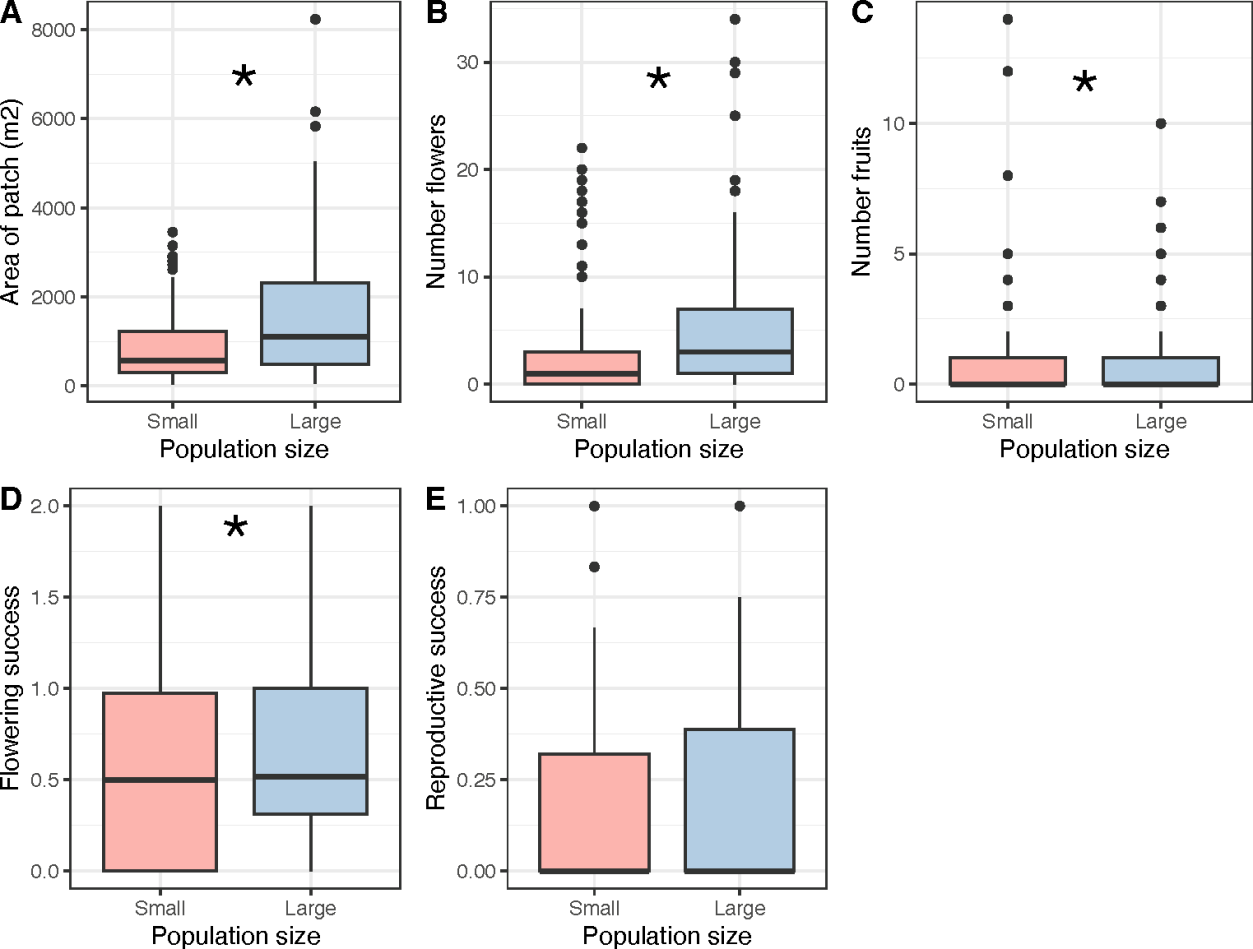
Plant fitness and reproduction traits. Boxplots showing (A) *Cypripedium calceolus* individual patch area, (B) number of flowers per plant, (C) number of fruits per plant, (D) flowering success (stems per flowers) and (E) reproductive success (fruits per flowers) in small (<20 individuals, red boxplots) and large (>100 individuals, blue boxplots) populations. Boxplots illustrate the median (middle bar) and the range between 1st and 3rd quartiles with the whiskers extending to the upper and lower inner values. Asterisks indicate significant differences across population sizes (*p* < 0.05).

We found that large populations of *C. calceolus* had, on average, 78.67% larger individuals (patch size) (figure 3A; population size effect: χ^2^_1,253_ = 25146, *p* = < 0.001, site effect: χ^2^_32,221_ = 82646, *p* = < 0.001), produced 92.90% more flowers per individual (figure 3B; population size effect: χ^2^_1,253_ = 108.47, *p* = < 0.001, site effect: χ^2^_32,221_ = 580.96, *p* = < 0.001) and had a flowering success (figure 3D; population size effect: χ^2^_1,253_ = 1.611, *p* = 0.028, site effect: χ^2^_32,221_ = 28.822, *p* = < 0.001) significantly more important than small populations. By contrast, we found no difference, for both the per capita number of fruits (figure 3C; population size effect: χ^2^_1,253_ = 4.055, *p* = 0.136, site effect: χ^2^_32,221_ = 235.700, *p* < 0.001) and the reproductive success of large *versus* small populations (figure 3E; population size effect: χ^2^_1,253_ = 0.005, *p* = 0.918, site effect: χ^2^_32,221_ = 47.214, *p* < 0.001).

### 3.3. Flower VOCs

Across the 85 *C*. *calceolus* individuals studied, we recorded a total of more than 40 compounds produced by *C. calceolus* flowers. To avoid biases due to interannual variation, out of the total number of VOCs observed, we selected 21 VOCs that were present across all three years of sampling (table 1). Of those, five were terpenoids (α-farnesene, limonene, linalool, u-sesquiterpene1, u-sesquiterpene2), twelve were aliphatic compounds (octanal, octanol, nonanal, decanal, octyl acetate, decyl acetate, (Z)-hexenol-acetate, hexyl acetate, lauryl acrylate, (Z)-7-hexadecenal, pentanoic acid, tetradecane), three were aromatic compounds (benzyl acetate, 2-phenyletanol, 2-phenetyl-acetate) and one was a cyclic ketone (4-oxoisophorone).

**Figure 4.**
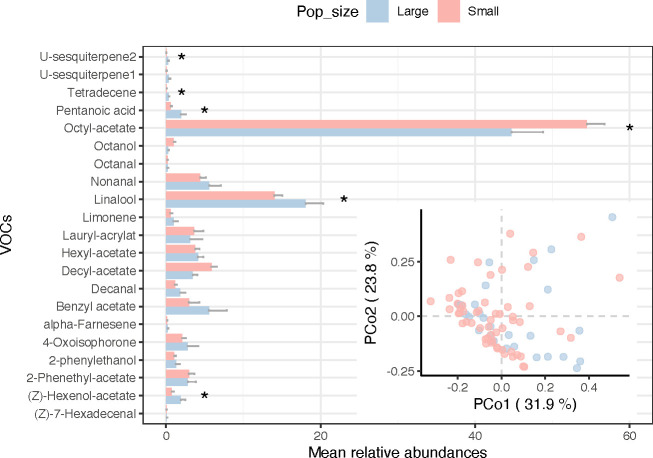
*Cypripedium calceolus* flower VOCs. Barplots (means ± s.e.) of mean relative abundance of each individual VOC found across small (<20 individuals, in red) and large (>100 individuals, in blue) populations. Inset shows the two-dimensional principal coordinate analysis (PCoA) for the VOCs. Asterisks indicate significant differences across population sizes (*p* < 0.05).

Both population size and site significantly influenced the variability in the chemical compound compositions among samples (figure 4; population size effect: *R*² = 0.027, *p* = 0.005, site effect: *R*² = 0.566, *p* = 0.001), in which six VOCs significantly differed between small and large populations (figure 4). Proportions of linalool (*p* = 0.038), (Z)-hexenol-acetate (*p* = 0.055), pentanoic acid (*p* = 0.009), tetradecene (*p* = 0.002) and u-sesquiterpene2 (*p* = 0.006) were higher in large populations. Only one VOC, octyl acetate, was significantly higher in small populations in average (figure 4; *p* = 0.029).

We found that only two chemical compounds have a significant effect on the prediction of the reproductive success. Among them, limonene showed a positive effect (electronic supplementary material, figure S5a; *p* = 0.030), whereas linalool (electronic supplementary material, figure S5b; *p* = 0.040) had a negative effect on reproductive success.

Finally, we found that *H. murorum* flowerheads produced a wide range of VOCs, including only four common compounds (octanal, (Z)-hexenol-acetate, nonanal and decanal) with *C. calceolus* (electronic supplementary material, figure S6a), while the overall VOC bouquets were significantly different between the two species (electronic supplementary material, figure S6b; PERMANOVA, *R*^2^ = 0.187, *p* = 0.001).

## 4. Discussion

Recent research has established that habitat fragmentation and degradation significantly threaten biodiversity [12], particularly endangering rare species [8,61]. On a broader scale, the loss of biodiversity can indirectly impact plant fitness, for example, by reducing pollinator populations when they are a limiting factor for survival [62,63]. Within this context, our study reveals that larger *C. calceolus* populations are associated with more diverse plant communities and a higher abundance and richness of insect pollinators compared to smaller populations. Furthermore, we found that flower odour chemistry varies with population size, with some compounds being consistently more abundant while others being consistently less abundant in small versus large populations, probably influencing plant–pollinator interactions [15,64]. Surprisingly, despite these differences, per capita reproductive success remains consistent across populations of different sizes. Below, we discuss our findings and explore potential explanations for this apparent paradox.

### 4.1. The cascading effect of habitat biodiversity on plant reproduction

It is assumed, and generally observed, that insect abundance positively correlates with vegetation diversity at the plot scale [65–68]. By providing a wider range of habitats and niches, more diverse vegetation communities favour the presence of a wider range of insect species and trophic groups [67,69]. Accordingly, we observed a positive association between vegetation diversity, species richness and abundance of potential pollinators, at least for some of the diversity metrics we examined. Two non-exclusive mechanisms may explain this pattern. First, large *C. calceolus* populations may establish in sites with more favourable conditions for vegetation growth, for example with richer soils, sufficient light reaching the soil and milder climatic conditions, which, in turn, promote both vegetation and insect diversity [30]. Second, larger populations may persist in areas that have experienced less habitat destruction and fragmentation than smaller populations, thereby supporting higher overall biodiversity [14,70]. At present, distinguishing between these two hypotheses remains challenging. We advocate for future work to investigate in more detail the links between surrounding vegetation diversity, insect diversity and population performance of rare species across a wide range of habitats.

Nonetheless, when looking at our insect surveys from a natural history point of view, we report six Hymenopteran species that are not yet known to be pollinators of *C. calceolus*. We also identified five new species of solitary bees and five species of syrphid flies that had not previously been reported to visit *C. calceolus* flowers (i.e. exiting the labellum but without pollinia). However, syrphid flies have already been shown to pollinate *C. calceolus* [23,24], or at least to act as flower visitors on this species [38,71,72]. Our results, therefore, confirm that the range of species pollinating this orchid is broad and made up of generalist pollinators, the majority of which are polylectic short-tongued solitary and eusocial bees [23,24,38], and that the list of known pollinators associated with this species is likely to be larger than what is described so far.

### 4.2. The conundrum of reproductive success according on pollinator diversity

Despite the greater diversity and abundance of potential pollinators in large *C. calceolus* populations, we observed that they produce, per capita, the same number of fruits and they have the same reproductive success as small populations. This occurred even though plants in small populations are smaller in size, produce fewer flowers and have less flowering success than large populations. These results might suggest that individuals from small populations, potentially growing in less suitable sites with for example poorer pollinator communities, attract as many pollinators as individuals in large populations. Indeed, we found no significant differences in insects trapped in the labellum of orchids between small and large populations. However, this result runs counter to some other evidence that pollination efficiency and fruit set are mainly a function of pollinator population size and density [73]. For instance, Albrecht *et al*. [74] reported enhanced pollination services with more diverse pollinator communities at the plant population level in a study of *Raphanus sativus*. These authors suggested that such a positive relationship is due to both the presence of specific taxa and niche complementarity in the pollinator community, such that greater pollinator diversity leads a greater number of fertilization events. Similarly, Agren [75] reported a positive correlation between *Lythrum salicaria* population size and seed set, suggesting that a pollinator deficit occurs in small plant populations due to insufficient pollen transfer. Finally, the reproductive success of the orchid *Listera ovata* has been shown to increase with population size [76], and some studies have shown that *C. calceolus* population size and pollination rate are positively correlated [36,77]. That said, our results are not unique, especially for our species. Another study on *C. calceolus* have shown a negative correlation between population size and reproductive success [78]. These results could be explained by the fact that *C. calceolus* is a rewardless species and reproductive success is thus reduced if pollinators learn to avoid it. This phenomenon is more likely to occur in large populations where patch size and number of flowers are greatest. This has been demonstrated in sexually deceptive orchids, where pollinators that have been deceived will rarely visited neighbouring orchids as a result of the deception [79]. Another explanation could be that large populations, which inhabit environments rich in plant diversity, experience greater interspecific competition for pollinators, thereby reducing the individual attractiveness of each plant [80–82]. Given these contrasting patterns in pollination dynamics, future research should aim to disentangle the relative influence of pollinator learning behaviour, interspecific competition, and habitat characteristics on the reproductive success of *C. calceolus*.

### 4.3. The variability in flower odours

In this study, we analysed the chemical composition of small and large populations of *C. calceolus* and found that they exhibit different chemical compositions. Indeed, the proportion of certain compounds varies between these populations. Octyl acetate, identified as a major VOC in *C. calceolus* and known to be highly attractive to its pollinators [24], is present in greater abundance in small populations than in large ones. Conversely, other compounds, such as linalool, (z)-hexenol-acetate, pentanoic acid, tetradecene and u-sesquiterpene2 are more prevalent in large populations. These findings suggest potential differences in the olfactory cues presented to pollinators across populations of varying sizes. A previous study on *C. calceolus* pollinators demonstrated that *Andrena* and *Lasioglossum* bees, as well as *Platycheirus* flies can respond to a wide range of VOCs through antennal detection [23]. However, our study did not directly measure pollinator behaviour, but rather identified correlations between the abundance of certain compounds and reproductive success. Specifically, linalool, which is more abundant in large populations, was negatively correlated with the reproductive success. This finding may help explain why populations in apparently favourable environmental conditions do not always exhibit higher fruit production. In contrast, limonene, which shows no significant differences between populations sizes, was positively associated with reproductive success, potentially contributing to the observed similarity in fruit production between small and large populations. Yet, it is important to emphasize that while our results reveal correlations between specific VOCs and reproductive success, they do not establish causal links. Other parameters may also influence pollinator attraction and reproductive outcomes, such as visual signals including the shape and colour of flowers for example [83,84]. Further studies integrating behavioural assays with pure compounds—of the correct chiral form—are necessary to fully understand the mechanisms underlying pollinator attraction and reproductive success in *C. calceolus*.

### 4.4. Linking reproductive success to co-flowering species

The reproductive strategies of *C. calceolus* individuals involve interactions with the surrounding floral environment, particularly through mechanisms that influence pollinator attraction. Two primary hypotheses could explain how this orchid benefits from co-flowering species: floral Batesian mimicry [85] and the magnet species effect [44]. Floral Batesian mimicry occurs when non-rewarding plants deceive pollinators by mimicking the sensory signals (e.g. scent, colour, shape) of rewarding species [42,86]. This deception leads pollinators to visit the mimic under the false expectation of a reward. However, our preliminary analysis of the VOCs of *H. murorum*, the most common co-flowering yellow species in our system, revealed no significant overlap with *C. calceolus*. This suggests that Batesian mimicry is unlikely in this context, as there is no clear chemical resemblance between the orchid and rewarding species that could lead to mistaken identity by pollinators. A more plausible explanation is the magnet species effect, which describes how non-rewarding plants benefit from pollinators already attracted to nearby rewarding species [44]. In this scenario, *C. calceolus* does not need to mimic a specific species; instead, it takes advantage of pollinators that are drawn to a diverse and abundant floral environment. This hypothesis could be tested by factorially removing colour mimics from a large and small populations, and measure pollinator attraction over time. Furthermore, the diverse floral scents emitted by surrounding plants may reduce the risk of pollinators learning to avoid *C. calceolus*. If pollinators fail to distinguish the orchid from the general floral environment, they may continue to visit it, even in the absence of a reward [87,88]. Interestingly, differences in VOC emissions between small and large populations of *C. calceolus* could reflect adaptations to varying floral community structures. We thus might predict that in smaller populations, where floral diversity is lower, orchids may rely more on their own scent cues to attract pollinators. Instead, in larger populations, where a richer floral environment exists, the orchid may benefit more from the magnet species effect by leveraging the chemical and visual diversity of surrounding vegetation. To fully understand the relative contributions of these mechanisms, further experimental research is needed. Future studies should investigate how pollinator behaviour varies in different floral contexts, whether *C. calceolus* benefits from specific co-flowering species, and to what extent floral community composition influences reproductive success.

## 5. Conclusions

Our findings indicate that floral VOC production differs between individuals from small populations, despite similar pollinator visitation rates in both small and large populations. We thus urge future work to focus on VOC production as a potentially major and general compensatory mechanism for the maintenance of plant reproductive fitness via plant–pollinator interactions. Such consideration is particularly important for rare plant species that grow in fragmented and pollinator-poor habitats, where reproductive fitness is likely to be low [89]. Moving forward, integrating floral chemical ecology into conservation planning could enhance the effectiveness of reintroduction and habitat restoration efforts. For instance, selecting individuals based on VOC profiles—particularly those associated with higher pollinator attraction—may improve the establishment and reproductive success of translocated populations. Understanding how floral scent variation interacts with landscape context and pollinator communities will be essential for ensuring the persistence of *C. calceolus* and similarly threatened species in fragmented ecosystems.

## Data Availability

Because of the sensitive nature of this dataset linked to an endangered species, the datasets generated during and/or analysed during the current study are available from the corresponding author on reasonable request. Supplementary material is available online [90].
